# Synergistic Contribution of HFE H63D Mutation to Secondary Polycythemia Pathogenesis at Moderate–High Altitude: A Retrospective Cohort Study

**DOI:** 10.3390/diagnostics16132087

**Published:** 2026-07-03

**Authors:** Tahir Alper Cinli, Ceren Alavanda

**Affiliations:** 1 Department of Hematology, Başakşehir Çam ve Sakura City Hospital, Istanbul 34480, Turkey; 2 Department of Medical Genetics, Istanbul University Faculty of Medicine, Istanbul 34093, Turkey; cerenalavanda@gmail.com

**Keywords:** polycythemia, erythrocytosis, HFE mutation, high altitude, hypoxia, iron metabolism, erythropoietin, hepcidin

## Abstract

**Objective:** This study examined whether HFE H63D carriers living at moderate-to-high altitude have a stronger secondary polycythemia phenotype than non-carriers from the same region. **Methods:** This retrospective cohort study included 59 patients with HFE gene mutation (heterozygous *n* = 49, homozygous *n* = 10) and 51 controls without HFE mutation (total *N* = 110). JAK2 V617F negativity was confirmed in all participants. Serum erythropoietin (EPO) levels were assessed in the HFE mutation group to support secondary, non-clonal erythrocytosis. **Results:** Hemoglobin (17.32 ± 1.29 vs. 15.11 ± 0.95 g/dL (*p* < 0.001), hematocrit (median 51.50 vs. 45.20; *p* < 0.001), and erythrocyte count (*p* < 0.001) were significantly higher in the HFE mutation group. Transferrin saturation was higher in the HFE mutation group than in controls (64.56 ± 2.03% vs. 36.30 ± 1.58%; *p* < 0.001; Cohen’s d = 0.95). Serum iron was higher and total iron-binding capacity was lower in the HFE mutation group, respectively (127.58 ± 44.10 vs. 93.62 ± 29.55 µg/dL and 194.32 ± 72.41 vs. 243.36 ± 40.82 µg/dL; both *p* < 0.001). CRP levels were also significantly elevated (*p* < 0.001). No significant differences were observed between the heterozygous and homozygous HFE mutation subgroup. **Conclusions:** HFE H63D carriers had higher erythrocytosis-related parameters and transferrin saturation than controls. These findings suggest that HFE-related changes in iron handling may strengthen the erythrocytosis phenotype at moderate-to-high altitude. Larger prospective studies should test this association.

## 1. Introduction

Polycythemia (erythrocytosis) is a clinical condition characterized by red blood cells increase. Today, it is classified in two major categories: primary (clonal, from the bone marrow) and secondary (erythropoietin-mediated) [1]. The diagnostic approach to differentiate primary polycythemia vera (PV) is based on screening for JAK2 V617F and JAK2 exon 12 mutations [2]. Chronic tissue hypoxia due to high altitude is one of the most common physiological and environmental causes of secondary polycythemia [3]. In the hypoxic environment, the hypoxia-inducible factor (HIF) pathway is activated in the peritubular cells of the kidney. This leads to compensatory erythrocytosis by increasing erythropoietin (EPO) synthesis [4,5,6]. In addition, the HIF pathway is important for the regulation of renal EPO production and intestinal iron absorption [7]. Genetic and metabolic factors may also influence this physiological response, as evidenced by the significant inter-individual variability in hematocrit levels found among residents at the same altitude [8]. EPO stimulation and sufficient iron in the bone marrow significantly affect erythropoiesis activity [9]. Mutations in the HFE gene, such as C282Y and H63D, suppress the body’s hepcidin production, thus leading to increased iron absorption from the intestine and faster release of iron from the reticuloendothelial system [10,11,12]. HFE protein is an MHC class I-like molecule that binds to the transferrin receptor in duodenal crypt cells. It is a key part of the system that keeps track of how much iron is in the body [13]. The C282Y mutation inhibits interaction of HFE protein with beta-2 microglobulin, and the H63D mutation reduces HFE-TfR1 interaction, which results in a moderate but clinically relevant disturbance of iron homeostasis [14,15]. Prospective cohort studies more recently have demonstrated that HFE gene variants are seen in 48–57% of patients diagnosed with idiopathic erythrocytosis, and subgroup analyses demonstrate that the H63D variant is especially common [16,17]. Current diagnostic frameworks for JAK2-unmutated erythrocytosis emphasize the importance of excluding congenital and secondary causes through a systematic clinical and molecular approach, particularly in patients classified as idiopathic erythrocytosis [18,19].

Experimental studies have demonstrated that the intestinal HIF-2α/iron metabolism pathway is pivotal in the onset of excessive erythrocytosis (EE) at high altitudes [20]. Under hypoxic conditions, the HIF pathway stops hepcidin from working, which speeds up the release of iron from the gut and macrophages and increases ferroportin activity [21,22]. The combined effect of increased availability of iron and chronic hypoxic stimulation at the same time on the erythropoietic response is not well understood. An important question that remains unanswered in the literature is whether subjects with HFE H63D mutation, in the context of chronic hypoxia in mid-to-high altitude regions, such as Van province at ~1700 m elevation, show a greater degree of secondary polycythemia than the general population [23,24]. This study tested this hypothesis in individuals living at moderate-to-high altitude.

## 2. Materials and Methods

### 2.1. Study Design and Ethical Approval

This study is a single-center retrospective cohort study that was conducted at Van Regional Training and Research Hospital. Ethical approval was obtained from the Ethics Committee of Van Regional Training and Research Hospital on 10 September 2025. (Ethics Committee No: GOKAEK72025-07-24). The study was performed in line with the principles of the Declaration of Helsinki. Given the retrospective nature of the study, informed consent was not required, but patient confidentiality and data security were fully maintained.

### 2.2. Participants

Two groups were included in the study: (1) 59 patients in whom HFE gene analysis was performed through the hospital information management system and who were found to carry the H63D mutation (heterozygous or homozygous); (2) a control group consisting of 51 individuals residing in Van province with no known HFE mutation. The total sample size was *N* = 110. Van province is located at approximately 1700 meters above sea level and is considered to be in the moderate-to-high altitude zone [25]. The exclusion criteria were as follows: positivity for JAK2 V617F; chronic cardiopulmonary diseases such as COPD and sleep apnea; active malignancy; chronic kidney disease (GFR < 60 mL/min/1.73 m^2^); active hematological disease; receipt of iron or EPO replacement therapy within the last six months; and inadequate medical record quality. The control group was selected from individuals who presented for routine health checkups or other reasons and were found to be negative for HFE mutation screening.

### 2.3. Data Collection

Demographic data, complete blood count parameters, iron metabolism parameters (serum iron, TIBC, transferrin saturation, and ferritin), liver and kidney function tests, inflammatory markers (CRP), nutritional parameters (vitamin B12, folate, and albumin), uric acid, and serum EPO levels were retrospectively obtained from the hospital electronic records system. Medical genetics reports were used to obtain HFE mutation status and JAK2 V617F results.

### 2.4. Statistical Analysis

Analyses were performed using IBM SPSS Statistics version 27.0 (IBM Corp., Armonk, NY, USA). Normality of distribution was assessed using the Shapiro–Wilk test; parametric data were presented as mean ± standard deviation, and non-normally distributed data were presented as median (interquartile range). Independent samples *t*-test (with Welch correction when homogeneity of variance was violated) and a Mann–Whitney U test were used for comparisons between two groups. Categorical variables were compared using the Pearson chi-square test or Fisher’s exact test. Effect size was calculated using Cohen’s d (≥0.80: large effect) [26]. To assess whether the relationship between HFE mutation status and erythrocytosis-related parameters was independent of ferritin level, separate ANCOVA models were applied for hemoglobin and hematocrit, with ferritin included as a covariate. The level of statistical significance was set at *p* < 0.05.

## 3. Results

### 3.1. Sociodemographic and Clinical Characteristics

**Figure 1:**
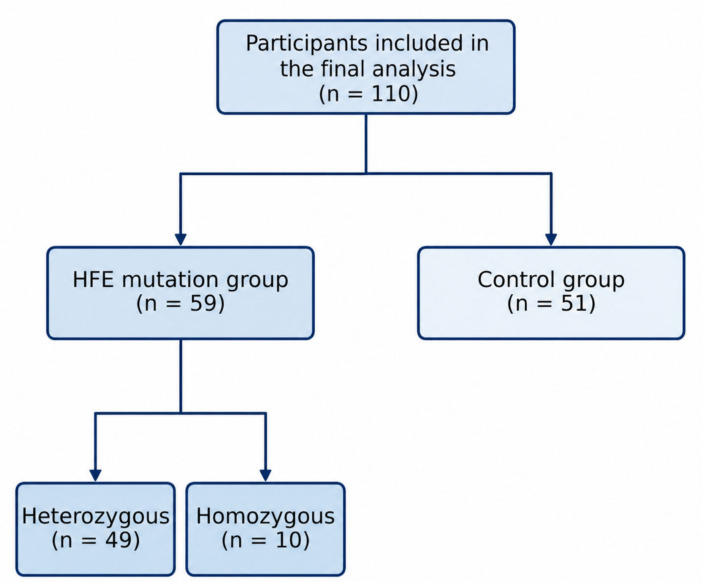
Flowchart of group allocation. The final analysis included 110 participants: 59 HFE mutation carriers and 51 controls.

The study included 110 participants, with 59 in the HFE H63D mutation group and 51 in the control group (Figure 1). In the mutation group, 49 patients were heterozygous and 10 homozygous. Two patients were heterozygous for the HFE H63D mutation and also carried the HFE C282Y mutation. JAK2 V617F negativity was confirmed in all patients, and clinical evaluation was consistent with secondary polycythemia; serum EPO levels also supported this finding (Table 1). No significant differences were observed between the groups in terms of age, sex, or smoking status (Table 1). The male sex was predominant in both groups. CRP levels were higher in the HFE mutation group, and this difference was statistically significant (*p* < 0.001) (Table 1). No significant differences were observed between the groups in terms of liver enzymes, uric acid, albumin, or creatinine levels (all *p* > 0.05) (Table 1).

### 3.2. Hematological Parameters

**Figure 2:**
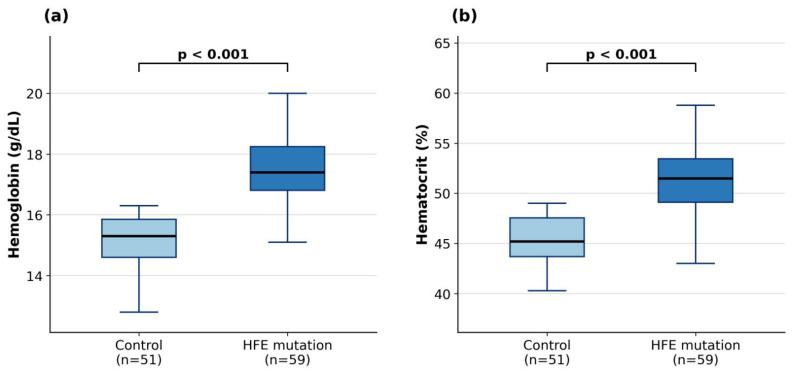
Comparison of hemoglobin and hematocrit levels between the *HFE* mutation and control groups. (**a**) Hemoglobin levels. (**b**) Hematocrit (HCT) levels. Box plots show median (bold line), interquartile range (box), and ). Both parameters were significantly higher in the HFE mutation group (*p* < 0.001).

Hemoglobin, hematocrit, and RBC levels were significantly higher in the HFE mutation group (Table 2 and Figure 2). Significant differences were also observed in erythrocyte indices: MCV, MCHC and RDW were all higher in the HFE group (Table 2). There were no significant differences between the groups in terms of White Blood Cell (WBC) count, neutrophils, or platelet count (Table 2). These results describe findings from this cohort. They should not be read as expected findings in all HFE H63D carriers.

### 3.3. Iron Metabolism Parameters

**Figure 3:**
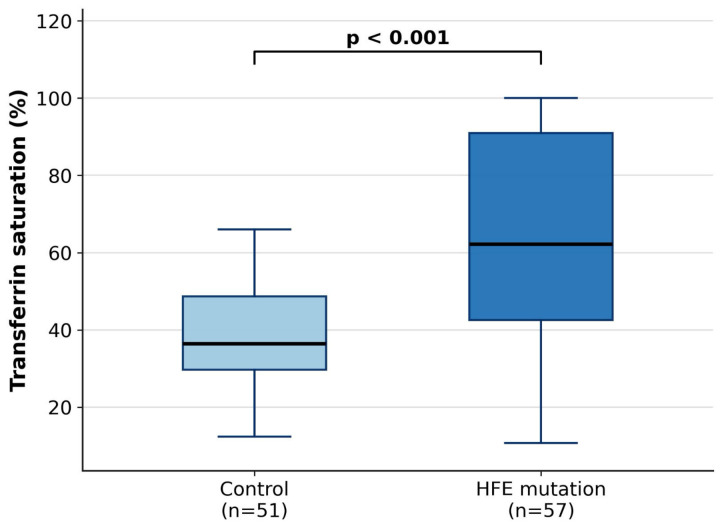
Comparison of transferrin saturation levels between the *HFE* mutation and control groups. The box plots illustrate the median, interquartile range, and range of transferrin saturation percentages for each study population. The HFE mutation group had higher transferrin saturation than the control group in this cohort (*p* < 0.001; Cohen’s d = 0.95).

Serum iron levels were higher in the HFE group than in the control group (Table 3). Total iron-binding capacity (TIBC) was significantly lower in the HFE group than in the control group (Table 3). Transferrin saturation showed the most striking difference between the two groups (Table 3; Figure 3). No significant difference was found between the groups in ferritin levels (Table 3).

### 3.4. HFE Mutation Subgroups: Comparison of Heterozygous and Homozygous Individuals

Among the 59 patients with the HFE H63D mutation, 49 were heterozygous, and 10 were homozygous. No statistically significant differences were observed between the two subgroups in terms of age, sex, hemoglobin, hematocrit, RBC, WBC count, platelets, erythrocyte indices, iron metabolism parameters, liver enzymes, CRP, or serum EPO levels (Table 4).

### 3.5. Ferritin-Adjusted Hemoglobin and Hematocrit Outcomes

Additional ANCOVA analyses were conducted to test whether the observed differences related to erythrocytosis were independent of ferritin levels. After controlling for ferritin as a covariate, both hemoglobin and hematocrit levels were significantly higher in the HFE mutation group than in controls (hemoglobin: F = 107.240, *p* < 0.001, partial η2 = 0.503; hematocrit: F = 64.330, *p* < 0.001, partial η2 = 0.378). Ferritin was not independently associated with hemoglobin (*p* = 0.227) or hematocrit (*p* = 0.119). These findings indicate that the observed erythrocytosis cannot be explained solely by the iron storage status.

## 4. Discussion

This retrospective cohort study demonstrated that HFE H63D mutation carriers living in Van province (~1700 m altitude) have a significant increase in hemoglobin, hematocrit and erythrocyte count as well as a marked impairment in iron metabolism compared with control individuals in the same geographic region. This study is a cohort study systematically comparing the erythropoietic activity of HFE H63D mutation carriers residing at moderate-to-high altitude with a control group.

The higher hematological indices in the HFE H63D group should not be viewed as an expected finding in all HFE H63D carriers. The H63D variant alone often has a modest and variable clinical effect. In this cohort, the higher hemoglobin, hematocrit, and red blood cell counts may reflect both HFE-related changes in iron handling and chronic hypoxic exposure at moderate-to-high altitude. For this reason, our findings should be viewed as hypothesis-generating and specific to this clinical setting.

### 4.1. Comparison with the Existing Literature: HFE Mutation and Erythrocytosis

In other studies, HFE mutation carriers were also found to have higher hemoglobin and hematocrit values. In a large retrospective cohort study of 152 HFE mutation carriers, Asif et al. reported a median hemoglobin level of 16.0 g/dL and hematocrit value of 47% in the H63D/H63D homozygous group [27]. These values were markedly lower than the mean hemoglobin level of 17.32 g/dL observed in the HFE group in our Van cohort. The most probable explanation for this discrepancy is that the study by Asif et al. was conducted at sea level, while our cohort was at an altitude of approximately 1700 m. This gives strong support to the hypothesis that the two effects, increased iron mobilization from the HFE mutation and hypoxia-induced stimulation of EPO, may be synergistic.

Abeyagunawardena et al. described a patient with heterozygous H63D and transferrin saturation of 61%, who developed erythrocytosis and iron overload after exclusion of clonal diseases (JAK2 V617F, MPL, CALR negative) [28]. The transferrin saturation levels in this case report were very close to the mean transferrin saturation level of 64.56% in the HFE group in our study. In our study, as in this case report, primary clonal hematological disorders were carefully ruled out and erythrocytosis was attributed to dysregulation of iron metabolism associated with the H63D mutation [28].

Data from cohorts with idiopathic erythrocytosis (IE) show an unexpectedly high prevalence of HFE mutations in this group. In a germline exome sequencing study of 56 patients with IE, Elli et al. found HFE-H63D or C282Y variants in 48.2% [16]. These findings suggest that HFE gene variants play a key role in the etiology of many “idiopathic” erythrocyts and are fully consistent with the main hypothesis of our study. Similarly, in a cohort of 118 JAK2-negative IE patients undergoing NGS panel analysis, Benetti et al. detected HFE gene variants in 57.1% of patients [17]. This is the highest mutation frequency of the single gene variants. These data support screening for HFE gene mutations in the routine work-up of JAK2-negative erythrocytosis.

### 4.2. High Altitude–Iron Metabolism Axis

At high altitude, the decreased availability of oxygen results in activation of the hypoxia-inducible factor (HIF) pathway and increased production of erythropoietin and erythropoiesis. HIF-2α also stimulates intestinal iron absorption and enhances iron availability for erythropoiesis by regulating the expression of the major iron transport proteins [4,7]. The HFE H63D mutation has been associated with altered iron homeostasis and decreased hepcidin activity. Thus, chronic hypoxic stimulation and HFE-associated iron regulatory abnormalities may act in concert to promote erythropoiesis. As a result, transferrin saturation was significantly higher in the HFE mutation group than in the controls (36.30% vs. 64.56%). Moreover, ANCOVA analyses indicated that the associations between HFE mutation status and both hemoglobin and hematocrit were significant after adjustment for ferritin levels. These results support that the observed erythrocytosis cannot be explained only by the iron storage status and the possible role of HFE-related abnormalities in the regulation of iron in the erythropoietic response under chronic hypoxic conditions.

### 4.3. Transferrin Saturation and Effect Size Findings

In the comprehensive erythrocytosis management guidelines updated by Gangat and Tefferi, it is recommended that transferrin saturation and iron parameters be included in the standard screening protocol for the evaluation of JAK2-negative erythrocytosis. In the most recent update published by the same group in 2025, this recommendation is further reinforced, stating that HFE mutations should be systematically investigated in the differential diagnosis of JAK2-negative erythrocytosis [29,30]. The lack of a significant difference in serum ferritin levels between the two groups (*p* = 0.606) suggests that the H63D mutation primarily affects the circulating iron compartment rather than iron stores, and in this context, ferritin may not be a sensitive marker of chronic iron loading in H63D carriers. This finding is consistent with the observations of Whitlock et al., who reported that iron overload is rarely observed in H63D/H63D homozygous individuals [31].

### 4.4. CRP Elevation and Inflammation–Erythropoiesis Interaction

The significant difference in CRP levels (*p* < 0.001) highlights the relationship between low-grade systemic inflammation and increased erythrocytosis. Increased iron load promotes the formation of reactive oxygen species (ROS), thereby creating a low-grade inflammatory environment [32]. However, the clinical implications of this finding require further investigation in prospective studies.

### 4.5. Clinical Significance of Heterozygous and Homozygous Subgroups

Hematological and biochemical parameters were not significantly different between heterozygous and homozygous H63D subgroups. This finding should be taken with caution, however, due to the limited sample size, particularly within the homozygous subgroup. The study may have been underpowered to detect genotype-dependent differences.

### 4.6. Clinical Implications and Screening Recommendations

The findings of this study suggest that, in individuals living at moderate-to-high altitude with JAK2-negative erythrocytosis, evaluation of iron parameters and consideration of HFE mutation analysis may provide additional diagnostic information. This approach is in line with contemporary recommendations advocating broader molecular assessment in unexplained erythrocytosis [18,19]. In the latest update guideline by Gangat and Tefferi, it is recommended that transferring saturation and HFE gene screening be integrated into the early-stage diagnostic algorithm for cases of JAK2-negative erythrocytosis [30]. Particularly in individuals residing above 1500 meters with unexplained erythrocytosis, the coexistence of iron profile abnormalities with HFE mutations should be considered an important clue to support the diagnosis of secondary polycythemia. The increasing role of hepcidin mimetics (e.g., rusfertide) in the treatment of polycythemia vera also offers a perspective that deserves further investigation in the context of the relationship between HFE mutations and erythrocytosis [33].

### 4.7. Limitations of the Study

The main limitations of this study can be summarized as follows: The retrospective design limits causal inference. HFE mutation screening was not performed in the control group as part of standard care, and the possibility of occult carrier status cannot be excluded . The sample size, particularly that of the homozygous subgroup (*n* = 10), limited the statistical power. Serum hepcidin measurements, erythroferrin (ERFE) levels, pulmonary function tests, and nocturnal oximetry data were not systematically recorded. Finally, the fact that the study is single-center and based on a specific geographic-ethnic population limits the generalizability of the findings. This hypothesis should be validated in larger cohorts through prospective multicenter studies incorporating hepcidin measurements and analyses of the HIF pathway.

## 5. Conclusions

This study demonstrates that the HFE H63D mutation interacts with chronic hypoxia at moderate-to-high altitudes (∼1700 m), leading to significant increases in hemoglobin, hematocrit, and erythrocyte count, as well as severe disturbances in iron metabolism (notably a large effect size in transferrin saturation). For individuals residing at moderate-to-high altitudes with unexplained erythrocytosis, iron profile assessment and HFE gene analysis should be incorporated into the standard diagnostic algorithm alongside JAK2 screening. Prospective multicenter studies are needed to generate data that will guide therapeutic management.

## Figures and Tables

**Table 1:** Comparison of sociodemographic and biochemical variables between groups.

Variable	Total *N* = 110	Normal Population *N* = 51	HFE Mutation Group *N* = 59	*p*
Age (years), Mean ± SD	38.90 ± 11.96	38.06 ± 8.75	39.64 ± 14.21	0.491 ^a^
Sex				
Female	17 (15.5%)	7(13.7%)	10 (16.9%)	0.641 ^b^
Male	93 (84.5%)	44 (86.3%)	49 (83.1%)	
Smoking Status				
No	92 (83.6%)	44 (86.3%)	48 (81.4%)	0.487 ^b^
Yes	18 (16.4%)	7 (13.7%)	11 (18.6%)	
Vitamin B12 (pg/mL), Median (IQR)	350.02 (134)	360.29 (151)	340.15 (98)	0.089 ^c^
Folate (ng/mL), Median (IQR)	6.80 (2.95)	6.55 (2.40)	7.35 (3.80)	0.270 ^c^
AST (U/L), Mean ± SD	19.11 ± 7.32	18.49 ± 7.34	19.66 ± 7.33	0.405 ^a^
ALT (U/L), Median (IQR)	20.0 (12.0)	19.0 (9.0)	20.0 (18.0)	0.668 ^c^
LDH (U/L), Median (IQR)	186.0 (48.0)	180.0 (31.0)	193.0 (52.5)	0.101 ^c^
GGT (U/L), Median (IQR)	20.0 (11.0)	20.0 (11.0)	19.5 (10.75)	0.937 ^c^
Alkaline Phosphatase (U/L), Mean ± SD	80.97 ± 21.87	77.76 ± 13.12	84.24 ± 27.92	0.142 ^a^
Uric Acid (mg/dL), Mean ± SD	5.50 ± 1.16	5.7 ± 1.13	5.29 ± 1.17	0.087 ^a^
EPO (mU/mL), Median (IQR)	7.93 (7.43)	—	7.93 (7.43)	—
Albumin (g/dL), Median (IQR)	4.62 (0.40)	4.60 (0.30)	4.80 (0.50)	0.199 ^c^
Creatinine (mg/dL), Mean ± SD	0.86 ± 0.14	0.89 ± 0.14	0.84 ± 0.14	0.052 ^a^
CRP (mg/dL), Median (IQR)	0.22 (0.32)	0.195 (0.284)	0.30 (0.45)	<0.001 ^c,^*

^a^: Independent samples *t*-test (with Welch correction); ^b^: Pearson chi-square test; ^c^: Mann–Whitney U test. * *p* < 0.05 is considered statistically significant. Mean: mean; SD: standard deviation; IQR: interquartile range; EPO: erythropoietin; CRP: C-reactive protein.

**Table 2:** Comparison of hematological parameters between groups.

Variable	Total *N* = 110	Normal Population *N* = 51	HFE Mutation Group *N* = 59	*p*
Hemoglobin (g/dL), Mean ± SD	16.29 ± 1.59	15.11 ± 0.95	17.32 ± 1.29	<0.001 ^a,^*
Hematocrit (%), Median (IQR)	47.90 (6.70)	45.20 (4.00)	51.50 (4.80)	<0.001 ^c,^*
RBC (10^6^/µL), Median (IQR)	5.41 (0.68)	5.20 (0.42)	5.77 (0.49)	<0.001 ^c,^*
WBC (/µL), Mean ± SD	7233.49 ± 1671.1	6977.15 ± 1678.9	7455.08 ± 1646.42	0.135 ^a^
Neutrophils (/µL), Median (IQR)	3895.0 (1637.5)	3850.0 (1150.0)	3900.0 (1970.0)	0.448 ^c^
Platelets (10^3^/µL), Mean ± SD	247.85 ± 52.1	247.47 ± 45.7	248.17 ± 57.3	0.944 ^a^
MCV (fL), Mean ± SD	87.92 ± 4.22	87.06 ± 3.67	88.68 ± 4.55	0.044 ^a,^*
MCHC (g/dL), Median (IQR)	33.6 (1.05)	33.40 (0.90)	33.90 (1.00)	0.005 ^c,^*
RDW (%), Median (IQR)	42.65 (3.95)	41.60 (4.0)	43.0 (3.30)	0.016 ^c,^*

^a^: Independent samples *t*-test (with Welch correction); ^c^: Mann–Whitney U test. * *p* < 0.05 is considered statistically significant. Mean: mean; SD: standard deviation; IQR: interquartile range.

**Table 3:** Comparison of iron parameters between groups.

Variable	Total *N* = 110	Normal Population *N* = 51	HFE Mutation Group *N* = 59	*p*
Ferritin (ng/mL), Mean ± SD	130 ± 1.4	123 ± 2.7	135 ± 1.77	0.606 ^a^
Serum Iron (µg/dL), Mean ± SD	111.54 ± 41.46	93.62 ± 29.55	127.58 ± 44.1	<0.001 ^a,^*
TIBC (µg/dL), Mean ± SD	217.47 ± 64.24	243.36 ± 40.82	194.32 ± 72.41	<0.001 ^a,^*
Transferrin Saturation (%), Mean ± SD	50.1 ± 1.95	36.30 ± 1.58	64.56 ± 2.03	<0.001 ^a,^*

^a^: Independent samples *t*-test (with Welch correction) * *p* < 0.05 is considered statistically significant. Mean: mean; SD: standard deviation; TIBC: total iron binding capacity.

**Table 4:** Comparison of heterozygous and homozygous subgroups in the HFE mutation group.

Variable	Heterozigot *N* = 49	Homozigot *N* = 10	*p*
Age (years), Mean ± SD	40.22 ± 14.98	36.8 ± 9.65	0.492 ^a^
Hemoglobin (g/dL), Mean ± SD	17.27 ± 1.34	17.59 ± 1.01	0.478 ^a^
Hematocrit (%), Median (IQR)	51.40 (5.50)	51.60 (6.38)	0.443 ^c^
RBC (10^6^/µL), Median (IQR)	5.78 (0.54)	5.73 (0.69)	0.664 ^c^
WBC (/µL), Mean ± SD	7422.04 ± 1745.13	7617 ± 1091.51	0.736 ^a^
Platelets (10^3^/µL), Mean ± SD	247.86 ± 60.47	249.7 ± 40.69	0.927 ^a^
MCV (fL), Mean ± SD	88.65 ± 4.25	88.82 ± 6.1	0.916 ^a^
MCHC (g/dL), Median (IQR)	34.0 (0.95)	33.75 (1.50)	0.856 ^c^
RDW (%), Median (IQR)	43.0 (3.50)	43.10 (4.05)	0.628 ^c^
Serum Iron (µg/dL), Mean ± SD	127.02 ± 44.41	130.2 ± 44.84	0.838 ^a^
TIBC (µg/dL), Mean ± SD	192.91 ± 77.68	200.9 ± 41.58	0.755 ^a^
Transferrin Saturation (%), Mean ± SD	64.56 ± 2.03	63.45 ± 1.89	0.784 ^a^
Ferritin (ng/mL), Mean ± SD	133.5 (175.25)	105.0 (262.75)	0.902 ^c^
EPO (mU/mL), Ort ± SD	5.56 ± 5.8	4.69 ± 0.46	0.657 ^a^
AST (U/L), Mean ± SD	19.53 ± 7.62	20.3 ± 5.96	0.765 ^a^
ALT (U/L), Median (IQR)	19.0 (17.50)	24.0 (21.75)	0.275 ^c^
Uric Acid (mg/dL), Mean ± SD	5.34 ± 1.21	5.08 ± 1.05	0.585 ^a^
Albumin (g/dL), Median (IQR)	4.8 (0.48)	4.63 (0.62)	0.852 ^c^
Creatinine (mg/dL), Mean ± SD	0.84 ± 0.15	0.82 ± 0.07	0.622 ^a^
CRP (mg/dL), Median (IQR)	1.28 ± 3.35	0.64 ± 0.54	0.216 ^a^

^a^ Independent samples *t*-test; ^c^ Mann–Whitney U test. Statistical significance was set at *p* < 0.05. Mean: mean; SD: standard deviation; IQR: interquartile range.

## Data Availability

The data presented in this study are available on request from the corresponding author. The data are not publicly available due to privacy and ethical restrictions related to patient confidentiality.

## Abbreviations

The following abbreviations are used in this manuscript:

HFE	Homeostatic Iron Regulator
EPO	Erythropoietin
TS	Transferrin Saturation
Hb	Hemoglobin
Hct	Hematocrit
CRP	C-reactive protein
JAK2	Janus Kinase 2
ROS	Reactive Oxygen Species
PV	Polycythemia Vera
MPN	Myeloproliferative Neoplasms
